# Diagnostic Procedures to Detect *Chlamydia trachomatis* Infections

**DOI:** 10.3390/microorganisms4030025

**Published:** 2016-08-05

**Authors:** Thomas Meyer

**Affiliations:** Institute of Medical Microbiology, Virology and Hygiene, University Medical Center Hamburg-Eppendorf (UKE), Hamburg 20246, Germany; th.meyer@uke.de; Tel.: +49(0)40-7410-52145; Fax: +49(0)40-7410-58420

**Keywords:** *Chlamydia trachomatis*, non gonococcal urethritis, cervicitis, pelvic inflammatory disease, lymphogranuloma, amplification tests, rapid diagnostic test, line assay, enzyme immunoassay, first void urine

## Abstract

The intracellular life style of chlamydia and the ability to cause persistent infections with low-grade replication requires tests with high analytical sensitivity to directly detect *C. trachomatis* (CT) in medical samples. Nucleic acid amplification tests (NAATs) are the most sensitive assays with a specificity similar to cell culture and are considered the method of choice for CT detection. In addition, NAATs can be performed on various clinical specimens that do not depend on specific transport and storage conditions, since NAATs do not require infectious bacteria. In the case of lower genital tract infections, first void urine and vaginal swabs are the recommended specimens for testing males and females, respectively. Infections of anorectal, oropharyngeal and ocular epithelia should also be tested by NAAT analysis of corresponding mucosal swabs. In particular, anorectal infections of men who have sex with men (MSM) should include evaluation of lymphogranuloma venereum (LGV) by identification of genotypes L1, L2 or L3. Detection of CT antigens by enzyme immunoassay (EIAs) or rapid diagnostic tests (RDTs) are unsuitable due to insufficient sensitivity and specificity. Recent PCR-based RDTs, however, are non-inferior to standard NAATs, and might be used at the point-of-care. Serology finds application in the diagnostic work-up of suspected chronic CT infection but is inappropriate to diagnose acute infections.

## 1. Introduction

Reports from the WHO indicate a world-wide increase of sexually transmitted infections (STI) in recent years, with *Chlamydia trachomatis* (CT) and Neisseria gonorrhoeae being the most frequent bacterial STI pathogens, each causing an estimated 106 million new infections per year [1]. In the USA, 1,441,789 chlamydial infections were reported in 2014, the highest number since cases were recorded in 1984. In the period of 2004 to 2014, the rate of reported chlamydial infection increased from 316.5 to 456.1 cases per 100,000 inhabitants [2]. Current data from the ECDC also demonstrate an increase of notified CT infections in Europe, rising from 191,000 in 2004 to 385,000 in 2013 and corresponding to an incidence of 162.8 and 181.8/100,000 inhabitants, respectively [3]. It is likely that the real number of new infections is even higher, since many infections are asymptomatic and remain undetected.

Several factors may account for the increase of diagnosed CT infections, including changes in sexual behavior and lack of prevention and education, but also more frequent testing with improved detection systems. In particular, sensitivity and specificity of STI testing was significantly enhanced by application of molecular techniques (nucleic acid amplification tests—NAATs). This review provides an overview of laboratory tests used to detect CT infections. The choice of tests and the diagnostic value of the selected procedure depend on the particular biologic characteristics of the pathogen and the clinical manifestation of CT infection, which will be briefly addressed first.

## 2. Pathogenesis, Genotypes and Clinical Manifestation

Chlamydia are obligate intracellular bacteria characterized by some unique properties associated with the intracellular lifestyle and the replication in host cells [4]. Whereas intracellular reticulate bodies (RB) represent the replicative form, extracellular elementary bodies (EB) act as infectious particles that target host cells via interaction of bacterial outer membrane proteins (MOMP, OmcB, PmpD) with host cell receptors, like heparan sulfate proteoglycans, mannose-6-phosphat-receptor and growth-factor receptors. After internalization, EBs are located in membrane-bound inclusion bodies expressing bacterial inclusion proteins that prevent fusion with lysosomes. Inclusion bodies were transported along microtubules to microtubule organizing centers (MTOC), where they differentiate into RBs. RBs replicate by binary fission and re-differentiate into EBs that will be liberated from inclusion bodies by cell lysis or extrusion and may infect other host cells [5].

The presence of chlamydia is recognized by the host organism by receptors of the innate immune systems, called pathogen/pattern recognition receptors (PRR). PRRs recognize particular structures of pathogens (pathogen associated molecular pattern—PAMP) and induce both innate and adaptive immune reactions mediating elimination of the pathogens. As the inflammation associated with the immune response is usually less pronounced, most infections remain asymptomatic. However, due to modification and subversion of various defense mechanisms (i.e. reduced antigen presentation and inhibition of expression of genes involved in cellular immunity) as well as induction of anti-apoptotic effects in infected host cells, chlamydia may potentially cause persistent infections. During the cellular immune reactions intracellular tryptophan levels decrease as a consequence of IFNy-induced indol dioxigenase. Chlamydia are auxotrophic for tryptophan and respond to this stress situation with generation of morphological aberrant, non-replicative, persistent forms that presumably convert into replicative forms as environmental conditions improve [4].

CT is divided into different serovars or genotypes that are associated with different clinical manifestations. Genotypes A–C are the dominant genotypes in ocular infections. Acute infections present as conjunctivitis that, if left untreated, may become chronic and lead to trachoma. These infections are rarely seen in Europe and North America, but are quite frequent in Africa and Asia, where they represent an important cause of blindness [6]. Genotypes D–K primarily cause infections of the urogenital tract, rectum, pharynx and conjunctiva [7]. In addition, perinatal transmission may cause conjunctivitis, pharyngitis and pneumonia in newborns [8]. Most rectal and pharyngeal infections and also many infections of the lower genital tract do not produce symptoms. However, both symptomatic and asymptomatic infections can cause sequelae, predominately affecting women. By ascending to the upper genital tract the bacteria may induce chronic inflammation resulting in pelvic inflammatory disease (PID), associated with a risk of ectopic pregnancy and tubal factor infertility (TFI) [9]. In order to reduce the rate of CT infections and complications many countries have introduced screening programs to identify asymptomatic infections through regular testing and to treat affected patients and their partners [10].

Whereas infections with genotypes A–K are normally confined to the mucosal epithelium, genotypes L1, L2 and L3 can cross the epithelium, spread via the lymphatics and cause invasive infections, called lymphogranuloma venereum (LGV). LGV is an endemic disease in parts of Africa, Asia, South America and the Caribbean, but until recently has rarely been found in industrialized countries. Since 2003 several outbreaks with genotype L2 were reported among MSM in Europe, North America and Australia [11]. These patients predominately suffer from anorectal symptoms, in contrast to the classical inguinal syndrome of sporadic LGV [12]. In 2013, more than 1000 cases were reported to the European Centre for Disease Prevention and Control (ECDC), about 50% of them from the UK [3]. The real number is likely to be higher, as in many European countries LGV is not routinely recorded. In addition, LGV infections are not always symptomatic, further contributing to the constantly high incidence rates [13].

## 3. Diagnostic Procedures

Testing for chlamydia is indicated in patients with urogenital, anorectal, and ocular symptoms, patients with STI other than chlamydia, sexual contacts of persons with STI, and persons destined for chlamydia screening [7]. Diagnostic procedures to detect CT infections include both direct and indirect methods. Generally, localized infections were examined by assays for direct pathogen detection, like culture, antigen tests (EIA, direct fluorescent antibody (DFA), and immune chromatographic RDTs), nucleic acid hybridization and amplification tests. Indirect methods depend on detection of antibodies against *C. trachomatis* that may be applied for diagnostic evaluation of chronic/invasive infection (PID, LGV) and post infectious complications, like sexually acquired reactive arthritis (SARA). In these conditions, pathogens have crossed the epithelial and may no longer be detectable in swabs. On the other hand, serology is inappropriate to diagnose acute infections of the lower genital and anal tract, as the antibody response becomes detectable only after weeks to months and is often less pronounced.

## 4. Isolation of *C. trachomatis* in Cell Culture

Established cell lines for isolation of *C. trachomatis* include Mc Coy, HeLa 229 or Buffalo Green Monkey Kidney cells. Swabs from different anatomical sites (endocervix, urethra, anal canal, conjunctivae) are suitable specimens for culture but must be collected using special devices and transport media [14]. Specimens were centrifuged onto confluent cell monolayer and analyzed for the development of characteristic intracytoplasmatic inclusions after 48–72 h by staining with Giemsa, iodine, or fluorescence labelled antibodies to chlamydial antigens (LPS or MOMP). When using MOMP-specific antibodies for staining cell culture, detection is highly specific [15], and thus it was long considered the reference test for CT detection. On the other hand, as culture depends on vital organisms the detection rate is at best 60%–80%, even when performed in laboratories with experienced technicians [16]. Sensitivity of culture may be impaired by inadequate specimen collection, storage and transport, toxic substances in clinical specimens and overgrowth of cell cultures by commensal microbes. Additional disadvantages are represented by the extended turn-around time, labor intensity and difficulties in standardization. Thus, cell culture is rarely used nowadays in diagnostic laboratories, but the methodology is still needed, at least in some reference laboratories, to monitor antibiotic susceptibility and changes of virulence, or when a test with the highest specificity is required as in case of suspected sexual assault.

## 5. Nucleic Acids Amplification Tests (NAATs)

NAATs are the most sensitive tests to detect chlamydia. These tests also have a high specificity comparable to culture, but in contrast to culture, do not depend on viable pathogens, facilitating specimen transport. Therefore, NAATs are generally considered the test of choice for chlamydia and have replaced culture as the diagnostic gold standard [14,17]. Antigen tests (EIA, DFA, RDTs) are no longer recommended for chlamydia testing due to insufficient diagnostic accuracy [14,17].

Most of the NAATs are based on polymerase chain reaction (PCR) und use fluorescence labelled probes to detect amplification products in real time, thereby significantly reducing the test duration. Combined with automated nucleic acid extraction, results can be generated in a few hours. In a number of studies, results of different NAATs were shown to be highly concordant [18,19]. Occasionally observed discordant results may relate to different analytical sensitivity, different efficacy of nucleic acid isolation and variability of chlamydia genomes [20]. The importance of genetic variation became evident with the appearance of the Swedish variant (*C. trachomatis* strain E/SW2) that was not detected by some commercial CT NAATs due to a deletion in the target region of these tests [21]. Moreover, gene regions may be exchanged by recombination when host cells were simultaneously infected with more than one CT strain [22,23]. Although the intracellular life style of chlamydia represents a rather high barrier for genetic recombination, genome sequencing of several CT strains indicate that horizontal gene transfer is not a rare event. Recombination primarily reflects adaptation to changing environmental conditions but may also result in development of new variants with increased virulence [23]. In addition, recombination is of relevance for laboratory diagnostics, especially when using tests based on nucleic acid detection. The implementation of a 2nd target region in NAATs (dual-target assays) represents an important improvement of NAATs, allowing detection of new variants with deletions or recombination in one of the target regions [24].

Diagnostic sensitivity was also enhanced by improved pre-analytical steps. Using coated magnetic beads nucleic acids were isolated in higher quantity and quality [20]. These bead-based extraction systems can be automated and are used in several high-throughput systems that allow simultaneous testing of chlamydia and gonococci with high sensitivity and specificity [25].

In populations with low prevalence of chlamydia, the predictive value of positive results is low, even when using tests with high specificity. For this reason, in 2002 the Centers for Disease Control and Prevention (CDC) has recommended confirming positive NAAT results with a second test in order to prevent unnecessary treatment and psychosocial consequences. Subsequent studies with improved NAATs have shown that non-confirmed positive NAAT results are not necessarily false positives, but may also represent false negative results of the confirmatory tests, especially when the latter test has a lower analytical sensitivity [26]. For samples with low amounts of chlamydia, the stochastic distribution of target DNA may result in some aliquots with concentrations below the limit of detection (LOD). Generally, confirmation of positive NAATs is not required and no longer recommended by the CDC. An important exception is represented by legal investigations in case of sexual assault. In order to avoid the serious consequences of false positive results in such cases, tests with the highest specificity are required. NAATs were shown to be more sensitive than culture to detect CT infections in victims of sexual assault [27], but when used for evaluation of sexual abuse, positive results must be confirmed with another NAAT using another target region [28].

## 6. Clinical Specimens for CT Testing

In principle, all relevant clinical materials can be analyzed by NAATs, including urethral, cervical, vulvo-vaginal, anorectal and ocular swabs, first void urine (FVU), sperm or tissues. FDA (Food and Drug Administration)-cleared commercial NAATs are approved for first void urine, urethral and cervical swabs, and most of them also for vaginal swabs [14]. Non-invasive specimens are preferred materials, in particular for screening of asymptomatic persons. FVU and urethral swabs from male patients are equivalent with respect to performance of CT NAATs. Collection of urine, however, is much better accepted and therefore the recommended sample type in men [17,29,30]. As the concentration of chlamydia sharply decreases during urination, it is important to use the first portion of micturition (approx 20 mL) [31]. In contrast, the chlamydia concentration in women with urogenital infection is comparatively higher in genital swabs than in urine [32]. A study analyzing urine, vaginal and cervical swabs taken simultaneously from asymptomatic women showed that the NAAT detection rate was highest in self-collected vaginal swabs [33]. Therefore, vaginal swabs (self-collected or clinician-collected) are the recommended sample type for women. Endocervical swabs may also be used, especially when a pelvic examination is indicated. FVU is less appropriate for CT testing in women, and when used for screening by NAAT, it should be considered that the detection rate is up to 10% lower compared to vaginal and endocervical swabs [14].

To detect extra-genital CT infections (conjunctivitis, anorectal or pharyngeal infections, incl. LGV) testing of corresponding swabs or tissue samples is required. CT infection of MSM is frequently localized in the rectum or pharynx without causing any symptoms. The majority of these infections would be missed when screening urine samples only [34,35], but require testing of appropriate oral and anal swabs to be diagnosed. Usually, commercial NAATs are not approved for testing non-genital sample types, but it has been shown in several studies that also in these specimens CT detection by NAATs is superior compared to culture or antigen tests [36,37,38,39]. Testing of specimens outside the approval of commercial tests requires evaluation of test performance characteristics according to quality assurance of microbiological diagnostics, such as CLIA (Clinical Laboratory Improvement Amendments). This also applies to testing of tissue samples by NAAT, as for instance endometrial samples or lymph node biopsies that may be considered in patients with PID or LGV, respectively. Furthermore, confirmation of LGV requires identification of genotypes L1, L2 or L3. LGV typing is important because the recommended antibiotic treatment is longer (Doxycyclin 200mg/day for at least 3 weeks in contrast to one week for non-LGV cases) [40]. Methods for typing include genotype-specific PCRs and RFLP or sequence analysis of appropriate omp1-gene regions [11,41,42]. LGV and non-LGV strains can also be differentiated by PCR tests based on a pmpH gene that in all LGV strains contains a 30bp deletion [42,43]. The pmpH PCR assay does not allow detection of specific genotypes. Sensitivity of the assay for LGV is high, but it misses about 25% of non LGV infections [44]. Thus, the assay is a suitable adjunctive test to identify LGV in CT positive samples, but it should not be used as a primary test [44].

## 7. Rapid Diagnostic Tests (RDTs)

Next to sufficient diagnostic accuracy of chlamydia tests, the time to generate and report test results is also important with respect to timely initiation of treatment. Usually, NAATs were performed in a central laboratory and require transportation of specimens and transmission of test results to the clinicians. Therefore, NAAT-based diagnostics requires a second visit of patients, potentially leading to delayed treatment or no treatment at all if patients do not re-appear again, which may contribute to the high incidence of infection. RDTs are independent of these demands on logistics, as they allow near-patient (point-of-care) testing and provide results in a few minutes, so that patients may receive antibiotic therapy immediately when they test positive. Most RDTs are immune chromatographic tests based on lateral-flow-technology and detect chlamydia LPS antigen in genital swabs or urine. Compared to culture and PCR these antigen-based RDTs are significantly less sensitive and less specific. In a study from Maastricht (The Netherlands) performed with self-collected vaginal swabs from 772 patients, the sensitivity of 3 chlamydia RDTs was 11.6%–27.3% compared to PCR as a reference test [45]. The low sensitivity of RDTs may relate to low bacterial load in asymptomatic patients, but even when testing endocervical swabs of symptomatic patients RDTs were only 22.7%–37.7% sensitive [46]. A better performance was reported for another chlamydia RDT developed at the University of Cambridge (CRT, Diagnostics for the Real World). Based on the combined data from 4 studies, the sensitivity for first void urine and vaginal swabs was 77% and 80%, respectively, each with a specificity of 99% [47]. However, these data were not confirmed in subsequent studies that reported sensitivities of 41.2% and 74.2% for vaginal swabs [48,49] and 41.4% and 20% for male FVUs [49,50]. Accordingly, the antigen-based RDTs were not recommended for CT testing of both asymptomatic (screening) and symptomatic patients.

In contrast to immune chromatographic RDTs for detection of CT antigen, molecular RDTs using nucleic acid amplification techniques have a high diagnostic accuracy, comparable to standard NAATs [51]. The Xpert assay of Cepheid was the first available commercial rapid NAAT that provides point-of-care testing of individual samples for CT and gonococci in approximately 90 min. The assay is based on real-time PCR carried out in a closed system. After application of the clinical sample to a cartridge, the subsequent steps of nucleic acid isolation, amplification and detection of PCR products proceed in a fully automated process. To achieve a high rate of immediate treatment initiation, patients must be willing to wait for 90 min [51]. Further molecular RDTs that generate results even more rapidly will be available shortly. The Io POC-test Chlamydia (Atlas Genetics), based on PCR in microfluidic systems and electrochemical detection of PCR products, produces results in about 30 min [52,53] and is approved in Europe since February 2016. Isothermal amplification techniques, like loop-mediated isothermal amplification (LAMP) or recombinase polymerase amplification (RPA) are more rapid than PCR and may further improve the use of NAATs at the point-of-care [54]. Of importance is that rapid diagnostic tests based on NAAT are subjected to quality assurance of microbiological diagnostics that are usually met by certified laboratories but are difficult to implement under point-of-care conditions.

## 8. Serology

Testing for chlamydia antibodies is not useful to diagnose local epithelial infections of the lower genital tract, because antibodies are detectable with a delay of several weeks, antibody titers may be low, and many serologic test are not able to differentiate antibodies against different chlamydia species. On the other hand, serology may be helpful in the diagnosis of chronic and invasive infections (PID, LGV, SARA). In most of these cases the bacteria are undetectable in anogenital swabs or urine, and serologic data may be used to evaluate causative chlamydia infection. As persistent CT infections and complications of ascending infections are usually associated with a positive antibody response, negative serology most likely rules out the involvement of chlamydia. On the other hand, positive serology represents no definite proof of associated chlamydia infection.

The microimmunefluorescence (MIF) test was long considered the reference method of chlamydia antibody testing. The MIF test has been used to diagnose neonatal *C. trachomatis* pneumonia. Serum IgM titer of 1:32 or greater is considered diagnostic of infection. In contrast, testing for IgG is not useful because they may represent passively transferred maternal antibody. Current guidelines from the Centers for Disease Control and Prevention (USA), the British Association of Sexual Health and HIV (BASHH) and the International Union against STI (IUSTI), however, do not recommend serologic testing to diagnose infant pneumonia. In addition, the MIF test is time-consuming and labor-intensive, and the reading of fluorescence signals is prone to subjective evaluation [55]. Therefore, enzyme immunoassays (EIA) and immunoblots or line assays are currently used more frequently to detect chlamydia antibodies [55,56]. Interpretation of serological results is affected by cross-reactivity within chlamydia species pathogenic for humans as well as non-pathogenic environmental species. Some EIAs based on detection of LPS antigen do not differentiate between different chlamydia species. Chlamydial LPS is generally considered a genus-specific antigen, but cross-reactivity with antibodies against LPS of other Gram-negative bacteria has been observed [57,58]. The diagnostic performance of chlamydia antibody testing was improved by using species-specific proteins or peptides. Immunogenic proteins of *C. trachomatis* identified by 2D PAGE [56] were used together with analogous proteins of C. pneumoniae and C. psittaci in a commercial line assay to allow differential evaluation of Chlamydia antibody reactivity.

Recently, a proteom array has been developed, containing GST fusion proteins representing 908 of the 918 known ORFs of the CT genome. Using this array, 27 immune dominant proteins were identified that were reactive with > 50% of human sera from patients with confirmed CT infection [59]. In another study the proteom-array was used to compare antibody profiles of patients with tubal factor infertility (TFI) and normal fertility [60]. Five proteins were identified that were reactive only with sera from TFI patients. Reactivity against these proteins was also detected in some sera from patients with acute infection, but these sera show additional reactivity against other proteins not observed in sera from patients with chronic infections. These results are promising, indicating different antibody reactivity in acute and chronic CT infection that might be used to characterize particular antibody panels as possible markers for different stages of infection [60].

## 9. Conclusions

*Chlamydia trachomatis* is an obligate intracellular bacterium that infects epithelial cells and fibroblasts. Due to interactions with a number of host factors and commensal microbes, replication in infected host cells varies considerably and may be very low in asymptomatic and persistent infections. Therefore, direct detection of *C. trachomatis* requires a test with high sensitivity. Of all diagnostic techniques, NAATs are the most sensitive tests. They also have a high specificity comparable to culture, and therefore represent the method of choice for *C. trachomatis* detection. Many different NAATs (commercial tests and in-house protocols) can be used in the laboratory and should be selected according to sample numbers, grade of automation and costs. In any case, the performance characteristics of the test should be evaluated according to quality assurance of microbiological diagnostics. As NAATs are usually performed in central laboratories, samples must be transported and communication of results is delayed, requiring a second visit of patients to receive antibiotic therapy. To immediately initiate treatment of positive patients, rapid diagnostic tests were developed that provide quick results, are easy to perform and can be used as point-of-care tests. Rapid tests that detect chlamydia antigens, however, have insufficient sensitivity and specificity. Novel rapid tests based on NAAT have much better performance, comparable to standard NAATs. These fully automated systems are independent of central laboratories and may improve the point-of-care testing for *Chlamydia trachomatis* infections in future.
